# Transcriptomic study of *Salmonella enterica* subspecies *enterica* serovar Typhi biofilm

**DOI:** 10.1186/s12864-017-4212-6

**Published:** 2017-10-31

**Authors:** Khee Chian Jason Chin, Todd Duane Taylor, Maxime Hebrard, Kogaan Anbalagan, Marjan Ganjali Dashti, Kia Kien Phua

**Affiliations:** 1 Institute for Research in Molecular Medicine, Universiti Sains Malaysia, 11800 Penang, USM Malaysia; 2 Laboratory for Integrated Bioinformatics, Core for Precise Measuring and Modeling, RIKEN Center for Integrative Medical Sciences, 1-7-22 Suehiro-cho, Tsurumi-ku, Yokohama, Kanagawa Japan; 3 USM-RIKEN Centre for Aging Science (URICAS), Universiti Sains Malaysia, 11800 Penang, USM Malaysia

**Keywords:** Biofilm, *Salmonella* Typhi, Transcriptome

## Abstract

**Background:**

Typhoid fever is an acute systemic infection of humans caused by *Salmonella enterica* subspecies *enterica* serovar Typhi (*S*. Typhi). In chronic carriers, the bacteria survive the harsh environment of the gallbladder by producing biofilm. The phenotype of *S*. Typhi biofilm cells is significantly different from the free-swimming planktonic cells, and studies have shown that they are associated with antibiotic resistance, immune system evasion, and bacterial persistence. However, the mechanism of this transition and the events leading to biofilm formation are unknown. High throughput sequencing was performed to identify the genes involved in biofilm formation and to postulate the mechanism of action.

**Results:**

Planktonic *S*. Typhi cells were cultured using standard nutrient broth whereas biofilm cells were cultured in a stressful environment using high shearing-force and bile to mimic the gallbladder. Sequencing libraries were prepared from *S*. Typhi planktonic cells and mature biofilm cells using the Illumina HiSeq 2500 platform, and the transcriptome data obtained were processed using Cufflinks bioinformatics suite of programs to investigate differential gene expression between the two phenotypes. A total of 35 up-regulated and 29 down-regulated genes were identified. The identities of the differentially expressed genes were confirmed using NCBI BLAST and their functions were analyzed. The results showed that the genes associated with metabolic processes and biofilm regulations were down-regulated while those associated with the membrane matrix and antibiotic resistance were highly up-regulated.

**Conclusions:**

It is proposed that the biofilm phenotype of *S*. Typhi allows the bacteria to increase production of the membrane matrix in order to serve as a physical shield and to adhere to surfaces, and enter an energy conservation state in response to the stressful environment. Conversely, the planktonic phenotype allows the bacteria to produce flagella and increase metabolic activity to enable the bacteria to migrate and form new colonies of infection. This data provide a basis for further studies to uncover the mechanism of biofilm formation in *S*. Typhi and to discover novel genes or pathways associated with the development of the typhoid carrier state.

**Electronic supplementary material:**

The online version of this article (10.1186/s12864-017-4212-6) contains supplementary material, which is available to authorized users.

## Background

Typhoid fever is an acute systemic infection of humans caused by *Salmonella enterica* subspecies *enterica* serovar Typhi (*S.* Typhi), a bacterium with a genome size of around 4.8 Mbp in length and containing at least 4900 annotated genes [1]. Typhoid fever is a human-specific disease and is endemic in many Asian countries, such as Malaysia and India, and also in many African countries [2, 3] where outbreaks of the disease frequently occur. Contraction of the disease in developed countries, such as the United States of America, is rare because of better access to clean water and higher standards of hygiene [4, 5].

During *S.* Typhi infection, the bacteria spread from the intestine via the blood and circulate throughout the body. The bacteria then invade various organs, such as the intestinal lymph nodes, liver, gallbladder and the Peyer’s patches, with patients frequently becoming chronic carriers [6]. While in the gallbladder, the bacteria forms a biofilm on either the gallstones or gallbladder epithelium, providing a stable environment for growth and survival of the bacteria, and resulting in increased resistance towards the host’s immune system and antimicrobial agents [7, 8]. The biofilm matrix consists of polysaccharide layers that form around *S.* Typhi to protect the bacteria from its harsh environment. It has been hypothesized that the transformation of this organism from the planktonic to the biofilm phenotype leads to persistence and development of the carrier state in man. Thus, understanding the mechanism of this process may provide avenues for developing therapeutic counter-measures for eradication of this disease.

Next generation sequencing (NGS) is a technology that allows researchers to determine whole genome sequences of organisms, transcriptomes and even perform epigenomics. To date there have been a limited number of studies on the transcriptome of bacteria and the genes involved in biofilm formation. A study conducted by Perkins and colleagues [1] on the transcriptome of *S.* Typhi grown under normal conditions identified numerous novel genes and small RNAs. However, this study, conducted using an Illumina-based sequencer, did not analyze the biofilm transcriptome.

In this study, the transcriptome of the *S.* Typhi mature biofilm phenotype was investigated and the genes that were differentially expressed were compared to the transcriptome of *S.* Typhi grown under normal conditions (planktonic phenotype).

## Methods

### Preparation of biofilm and extraction of RNA

In this study, *S.* Typhi strain ATCC 7251 was obtained from the Bio-bank, Institute for Research in Molecular Medicine, Universiti Sains Malaysia, Malaysia. Standard serological and biochemical testing were done to confirm the *S.* Typhi organism before subsequent preparations for transcriptome analysis.

*S.* Typhi planktonic cells were grown in 1% nutrient broth (HiMedia, India), prepared as per the manufacturer’s protocol, and incubated in a shaking incubator at 37 °C until an OD_600_ value of 0.6 was recorded on the UV spectrometer (Spectronic BioMate 3, ThermoFisher Scientific, USA). *S.* Typhi biofilm tissue was grown in 50 ml Falcon tubes (SPL Life Sciences, Korea) using an optimized method described by Ganjali Dashti et al. [9]. Briefly, to simulate the gallbladder environment, a biofilm media consisting of a combination of 1.22% ox-bile (HiMedia, India), 0.5 M sodium chloride (Bio Basic Inc., Canada), 0.001 M potassium chloride (Merck, USA), 0.56 M glucose (Bio Basic Inc., Canada) and 1% nutrient broth was used to culture the *S.* Typhi cells in 50 ml Falcon tubes. In the tube, 1 ml of planktonic cells was added to 24 ml biofilm media. The tubes were placed into an incubator shaker for 24 h at 37 °C with a rotation speed of 250 rpm. The presence of bile and application of strong agitation caused the *S.* Typhi cells to form biofilm tissues to protect themselves from the stressful environment. The sticky layer that formed and attached to the surface of the polypropylene tubes was considered as the mature biofilm and was collected for RNA extraction.

RNA extraction was conducted using TRIzol® (Life Technologies, USA). For RNA extraction of planktonic cells, 15 ml of *S.* Typhi culture grown in nutrient broth was collected and pelleted down using a centrifuge at 12,000 xg for 15 min at 4 °C. The supernatant was removed and the pellet was washed once with 1× phosphate-buffered saline to remove residual nutrient broth. TRIzol® was added to the pellet, the tissue was then homogenized, followed by nucleic acid extraction according to the manufacturer’s protocol. For biofilm tissues, a Bead beater (Biospec Products, USA) was used to disrupt the polysaccharide matrix of the biofilm tissue. A total of 0.1 g of biofilm tissue was collected in a 2 ml centrifuge tube pre-filled with 500 mg zirconium beads (Benchmark, USA). The mixture was then homogenized at a revolution rate of 4800 rpm for 10 s with 30-s intervals cooling in ice for a total of 30 s of homogenization time. For each sample, three replicates were carried out to ensure complete homogenization of the biofilm tissue in TRIzol®. The quality of extracted RNA for both planktonic and biofilm cells was checked using an Agilent Bioanalyzer 2100 (Agilent Technologies, USA), and the concentrations were determined using a Nanodrop 2000c spectrophotometer system (Thermofisher, USA). Prior to cDNA conversion, the extracted RNA from planktonic and biofilm *S*. Typhi cells were treated with DNase I to remove genomic DNA contamination.

### RNA-sequencing

RNA-sequencing was conducted using an Illumina HiSeq 2500 machine. The extracted RNA samples from both planktonic and biofilm *S.* Typhi cells were subjected to the Illumina sequencing workflow by the service provider [10]. The workflow included ribosomal RNA depletion, cDNA conversion, library preparation and cluster generation followed by sequencing. Efficient library generation was then assessed using a Bioanalyzer platform (Agilent), and an Illumina MiSeq-QC run was performed. A total of three replicates were sequenced for each sample type. Paired-end transcript reads of 100 bp length with coverage of around one million transcript reads per sample were generated. The raw transcript reads were kept and stored in separate files per sample in “fastq” file format. Data files were up-loaded to at the NCBI Sequence Read Archive (SRA) (GEO Accession Number: GSE93686).

### Differential expression analysis

To acquire the counts data for differential expression analysis of the data generated, the two sample types, i.e. planktonic cells and biofilm cells, were mapped to the reference genome *S.* Typhi CT18 downloaded from the NCBI RefSeq database (Accession number: NC_003198.1). Data processing was done using the Tuxedo Suite program installed on a bioinformatics server at RIKEN. First Bowtie2 (Version 2.2.6) [11] was used to align the transcripts against the reference genome to produce a SAM file. The SAM file was then converted to BAM format and sorted by genome coordinates using SAMtools (Version 0.0.18) [12]. After sorting, Cufflinks (Version 2.2.1) was used for calculating the differential gene expression levels between the planktonic cells and biofilm cells [12]. CummeRbund (Version 2.12.1), an R program written for Cufflinks, was used to view and tabulate the results in an Excel file [13]. Gene annotation was also done using CummeRbund. Gene function information was added to the tabulated Cuffdiff results using an in-house Perl script.

## Results

A total of 141,883,512,100 bp paired-end reads were generated by the Illumina HiSeq 2500 platform. The reads were then subjected to Illumina sequencing adapter trimming and base quality (Q ≥ 20) trimming using Fastq-MCF (version 1.04.636) [14] to ensure that only high quality bases derived from the mRNAs were used for subsequent analysis. Trimmed reads less than 70 bp in length were discarded along with their mate-pairs. The overall alignment rate of all replicates was above 95%, showing good quality of the data obtained. A summary of the data is shown in Table 1.

**Table 1:** Total number of reads and overall percentage of reads mapped to the reference genome

Sample	Overall Mapped Reads (%)	Sequenced Reads
Planktonic		
Planktonic Replicate 1	97.53	20,829,742
Planktonic Replicate 2	97.76	25,992,472
Planktonic Replicate 3	97.12	23,777,118
Total Reads		70,599,332
Biofilm		
Biofilm Replicate 1	97.75	26,563,886
Biofilm Replicate 2	95.60	20,220,668
Biofilm Replicate 3	97.93	24,499,626
Total Reads		71,284,180

**Fig. 1:**
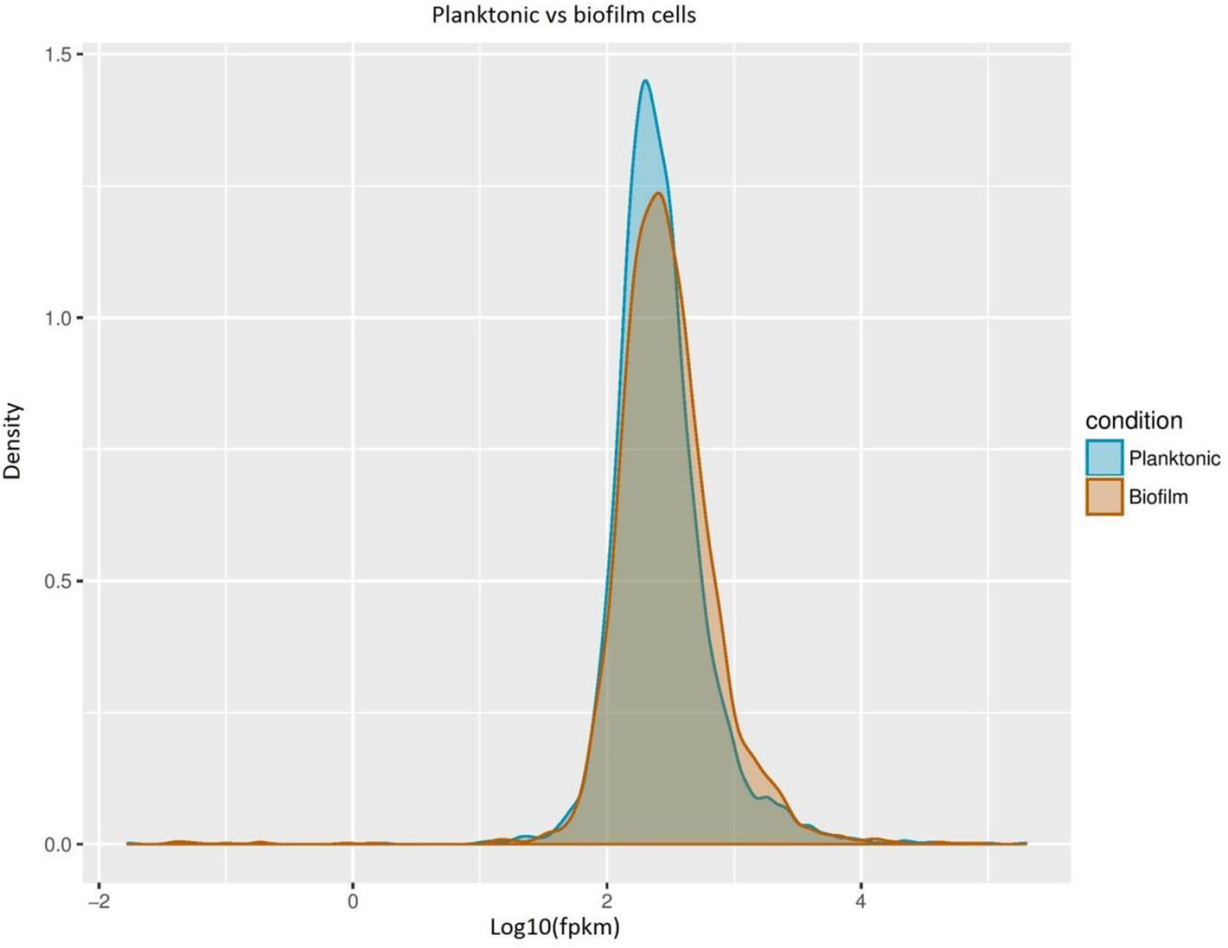
Density graph showing the distribution of expression levels for each sample type

Figure 1 shows the density of the transcript reads for both the planktonic and biofilm cells. The planktonic cells appear to have more genes that were expressed within the log_10_ (FPKM) value of 2 to 3 compared to the biofilm cells that had a lower expression value in the same range. However, the biofilm cells have higher gene expression levels as most of the reads were skewed towards the log_10_ (FPKM) value of 3 to 4. Overall, around 90% of the gene expression levels were similar between the two sample types.

**Fig. 2:**
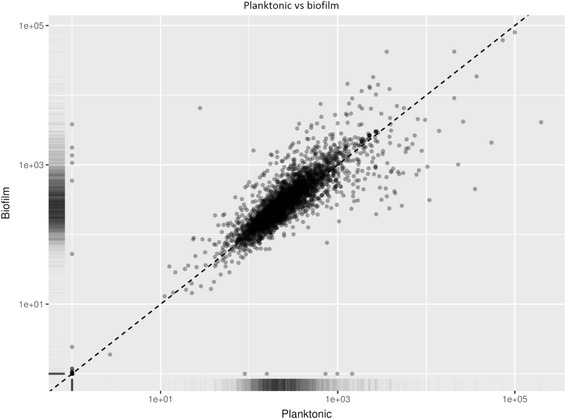
Scatter plot of the FPKM reads for both the planktonic and biofilm cells

Figure 2 is a scatter plot showing the relationship of the genes for both the planktonic and biofilm phenotypes. There are some outliers, but overall both samples have a positive relationship between gene expression levels. The outliers are considered the genes of interest as they show the most different up- or down-regulated genes between the two sample types due to the different growth environments.

**Fig. 3:**
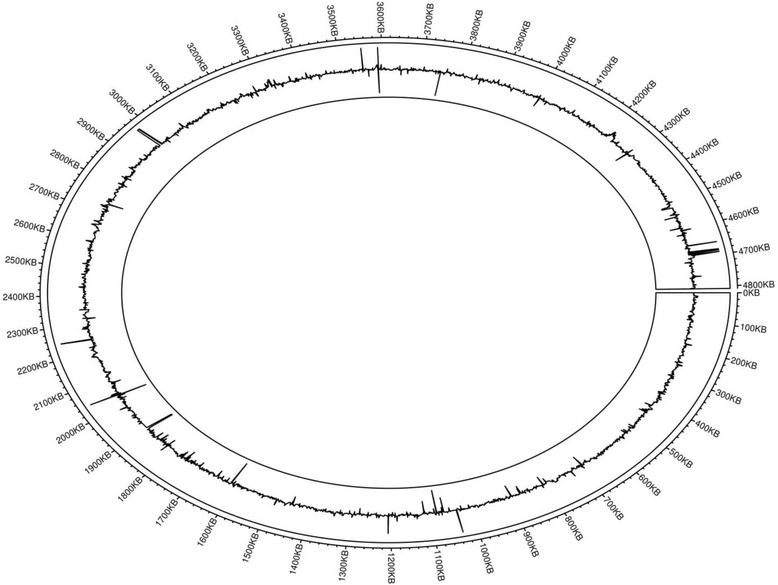
Circular plot of the reads mapping to the *S.* Typhi CT18 genome. The outer circle shows the genomic track, marked in 100 kb increments. The peaks in the track represent the log_2_-fold change of each gene, e.g. up-regulated genes *STY1254* (1,207,090...1207347), and *yheA* (4,231,386...4231580), and down-regulated genes *rmf* (1,066,679...1066846) and *STY1156* (1,116,294...1116461)

To help visualize the differentially expressed genes of *S.* Typhi planktonic and biofilm cells, the data from the transcriptome analysis was plotted using the R package Circlize software (Version 0.3.8) (Fig. 3) [15]. To generate the differential expressed gene map, the log_2_-fold change of each gene is mapped at its gene position on the genome track. This allowed for both the overall data (both significant and non-significant data) to be viewed. This data enables us to determine if there are any zones that have genes that were highly up-regulated or down-regulated. For example, in the area between 4660 kb and 4700 kb there were four highly up-regulated genes; *pyrL* (4,661,019…4,661,120), *STYt076* (4,683,605...4683689), *STY4824* (4,687,302...4688465) and *STY4832* (4,692,212...4694545), while in the region between 1600 kb and 1900 kb there were numerous down-regulated genes, such as *lppA* (1,666,834...1667070), *osmE* (1,721,748...1722089), and *STY1854* (1,764,423...1764671). The analysis of the results focused on the genes that were found to have statistically different levels of expression between the planktonic and biofilm cells. A total of 341 genes were found to be statistically significant (q-value <0.05). Among the 341 genes, only genes with log_2_-fold change values of greater than 2 and less than negative 2 were selected. There were a total of 35 up-regulated genes and 29 down-regulated genes. Data for selected genes are shown in Tables 2 and 3 for the up-regulated and down-regulated genes, respectively. For the full list of genes that were significantly up or down regulated, please refer to Additional file 1.

**Table 2:** Selected differentially up-regulated genes and their functions in *S.* Typhi biofilm cells

Gene	Gene function	log_2_-fold change	*p*-value	q-value
*STY1254*	Hypothetical protein	7.92174	5.00E-05	0.00183687
*STY1255*	Hypothetical protein	3.85549	0.00045	0.00997957
*yheA*	Bacterioferritin-associated ferredoxin	3.55635	2.00E-04	0.00568281
*rplD,rplW*	50S ribosomal subunit protein L4, 50S ribosomal subunit protein L23	3.50218	7.00E-04	0.0133293
*STY3469*	Hypothetical protein	3.41257	5.00E-05	0.00183687
*priB*	30s ribosomal subunit protein S18	3.25938	2.00E-04	0.00568281
*rpsS*	50S ribosomal subunit protein L22	2.93416	5.00E-05	0.00183687
*STY4905*	Hypothetical protein	2.8976	5.00E-05	0.00183687
*rplU*	50S ribosomal subunit protein L21	2.76352	5.00E-05	0.00183687
*rpmA*	50S ribosomal subunit protein L27	2.74781	5.00E-05	0.00183687
*rpsJ*	50S ribosomal subunit protein L3	2.65265	5.00E-05	0.00183687
*STY1229*	Hypothetical protein	2.60152	2.00E-04	0.00568281
*lexA*	LexA repressor	2.58813	5.00E-05	0.00183687
*rplV*	30S ribosomal subunit protein S3	2.53445	0.00015	0.00458445
*rpsI*	30S ribosomal subunit protein S9	2.51349	5.00E-05	0.00183687
*rplR*	30S ribosomal subunit protein S5	2.4115	5.00E-05	0.00183687
*rpsF*	30s ribosomal protein S6	2.41089	5.00E-05	0.00183687
*yhdG*	tRNA-dihydrouridine synthase B	2.38131	1.00E-04	0.00324732
*rplK*	50S ribosomal subunit protein L11	2.35434	5.00E-05	0.00183687
*marA,marR*	Multiple antibiotic resistance protein MarA, multiple antibiotic resistance protein MarR	2.33256	0.00105	0.0185381
*pdhR*	Pyruvate dehydrogenase complex repressor	2.32856	5.00E-05	0.00183687
*rplM*	50S ribosomal subunit protein L13	2.32687	0.00135	0.022217
*STY4034*	Putative IS1351 transposase (pseudogene)	2.30975	0.00125	0.0211453
*ygbA*	Hypothetical protein	2.27833	5.00E-05	0.00183687
*yfhP*	DNA-binding transcriptional regulator IscR	2.27551	0.00065	0.0127099
*rplA*	50S ribosomal subunit protein L1	2.24644	0.00205	0.0288986
*hmpA*	Flavohemoprotein	2.19086	9.00E-04	0.0162045
*rpsN*	30S ribosomal subunit protein S8	2.18809	5.00E-05	0.00183687
*rplB*	30S ribosomal subunit protein S19	2.13394	2.00E-04	0.00568281
*yejG*	Hypothetical protein	2.06615	5.00E-05	0.00183687
*STY1389*	Cyclic-di-GMP-binding biofilm dispersal mediator protein	1.79388	0.00365	0.0443982

**Table 3:** Selected differentially down-regulated genes and their functions in *S.* Typhi biofilm cells

Gene	Gene function	Log_2_-fold change	*p*-value	q-value
*STY1856*	Regulatory protein	−2.07402	5.00E-05	0.001837
*yiiU*	*FtsZ* stabilizer	−2.10309	5.00E-05	0.001837
*cspB*	Cold shock protein	−2.18312	5.00E-05	0.001837
*STY0398*	Propionate catabolism operon regulatory protein	−2.29804	5.00E-05	0.001837
*STY0788*	Hypothetical protein	−2.35255	5.00E-05	0.001837
*osmE*	Osmotically inducible lipoprotein E	−2.36034	5.00E-05	0.001837
*tatE*	Sec-independent protein translocase protein *TatE*	−2.41698	5.00E-05	0.001837
*ompX*	Outer membrane protein X	−2.45886	2.00E-04	0.005683
*yaiA*	Hypothetical protein	−2.48198	5.00E-05	0.001837
*hupA*	Histone like DNA-binding protein HU-alpha	−2.59176	5.00E-05	0.001837
*STY1982*	Hypothetical protein	−2.61628	5.00E-05	0.001837
*STY1323*	Hypothetical protein	−2.67081	5.00E-05	0.001837
*ompC*	Outer membrane protein C	−2.75117	5.00E-05	0.001837
*lppA*	Major outer membrane lipoprotein	−2.93078	5.00E-05	0.001837
*STY1938*	Hypothetical protein	−3.30784	5.00E-05	0.001837
*rpsV*	30S ribosomal protein S22	−3.30884	5.00E-05	0.001837
*yjfO*	Biofilm stress and motility protein A	−3.3313	5.00E-05	0.001837
*cspC*	Cold shock-like protein *CspC*	−3.34324	5.00E-05	0.001837
*STY2264*	Hypothetical protein	−3.38655	5.00E-05	0.001837
*dps*	DNA protection during starvation protein	−3.42148	5.00E-05	0.001837
*STY4154*	DNA-binding protein	−3.45538	0.00085	0.015693
*ecnB*	Entericidin B	−3.5082	5.00E-05	0.001837
*STY0800*	Hypothetical protein	−3.80587	5.00E-05	0.001837
*STY1854*	Hypothetical protein	−4.01921	5.00E-05	0.001837
*cspE*	Cold shock-like protein *CspE*	−4.36039	5.00E-05	0.001837
*STY4436*	Hypothetical protein	−4.50574	5.00E-05	0.001837
*STY0893*	Biofilm formation regulatory protein bssr	−5.12497	5.00E-05	0.001837
*rmf*	Ribosome modulation factor	−6.30581	5.00E-05	0.001837

The results were validated using Real-Time PCR. Please refer to Additional file 2 for detailed Real-Time PCR results.

## Discussion

The up-regulated genes include 3 hypothetical proteins and 18 ribosomal subunit genes. The down-regulated genes include 9 hypothetical proteins. NCBI BLAST analysis showed that the gene *STY4905* was similar to gene *DUF1435*, a putative membrane protein in *Salmonella enterica* subsp. *enterica* serovar Senftenberg. Gene *STY1229* was found to code for a protein which was closely related to ribosomal protein L32p in *Salmonella enterica* subsp. *enterica* serovar Weltevreden. The gene *STY3469* had no orthologs, which suggests that it is a unique protein associated with *S*. Typhi biofilm genesis.

In the down-regulated group, only 2 genes were identified; *STY1854* and *STY2264*. NCBI BLAST showed that *STY2264* was an ortholog of the gene *yeeX*, found in *Salmonella enterica* subsp. *enterica* serovar Typhimurium strain YU15. The gene *STY1854* was identified to be a histidine kinase protein unique to *Salmonella enterica* subsp. *enterica* serovar Typhi. The other 7 genes were unknown and did not have any orthologs.

For a deeper analysis of the possible functions of the genes in their role for biofilm formation in *S.* Typhi, the genes were grouped according to the following categories: 1. Genes responsible for the bacteria membrane matrix, 2. Genes responsible for antibiotic resistance, 3. Genes with general metabolic functions, and 4. Genes involved in biofilm regulation.

### Genes responsible for bacteria membrane matrix

The gene that was most highly up-regulated in the biofilm cells was *STY1254*, a multiple stress resistance protein, *bhsA*. It plays a role in the membrane structure of *S.* Typhi [16]. According to a study by Zhang et al. [17], the *bhsA* gene increases the stickiness of the membrane protein in *E. coli*, allowing it to stick to surfaces during biofilm formation. The researchers showed that deletion of the *bhsA* gene in *E. coli* caused more biofilm to form as the bacteria was not able to stick to the apparatus surface, causing them to form more biofilm matrix for protection. Gene *STY1255* is involved in the peptidoglycan biosynthesis pathway and is part of the cell wall biogenesis [18]. It also plays a part in anchoring the major outer membrane Braun lipoprotein to the peptidoglycan to stabilize the cell wall, giving biofilm cells a thicker cell wall and added stability [19].

Based on the functions of the genes *STY1254* (*bhsA)* and *STY1255*, and the results from our transcriptome study, the two genes may be responsible for the binding of *S.* Typhi to the surface of the polypropylene tubes, thus allowing biofilm formation and maintenance. Not only were the two genes responsible for adhesion, they also affected the properties of the surface membrane, which presumably allows the bacteria to survive the acidic environment of the biofilm culture media [16–19]. It was also found in a study by Salazar et al. [20] that *ycfR*/*bhsA* promotes the attachment of *S*. Typhimurium to the surface of glass, polystyrene, spinach leaves and tomato fruit.

The gene *STY1389*, which codes for cyclic-di-GMP-binding biofilm dispersal mediator protein, was also found to be highly up-regulated in mature biofilm (log_2_-fold change = 1.79). In a review paper by Valentinin and Filloux [21], it was shown that cyclic-di-GMP regulated biofilm formation in *P. aeruginosa* by affecting various pathways such as flagella rotation to Type IV pili retraction, exopolysaccharide production, surface adhesin expression, antimicrobial resistance, and biofilm dispersion. Our result suggests that the gene *STY1389* is responsible for mediating biofilm dispersion and propagation of infection at the mature stage of the biofilm cycle.

### Genes responsible for antibiotic resistance

Based on the transcriptome data, the Mar regulon genes, *marA* and *marR*, were significantly up-regulated in the biofilm cells as compared to the planktonic cells. Perera and Grove [22] indicated that the Mar regulon was associated with antibiotic resistance and stress responses. The Mar regulon also regulates bacterial virulence factors, such as cell wall proteins and surface adhesins, as reported by Prieto and colleagues [23] which showed that the genes *marA* and *marB* were up-regulated when exposed to bile. Thus, it can be hypothesized that the Mar regulon prepares the *S.* Typhi cells for potential antibiotic resistance, or against stress factors, such as the bile used in growing the biofilm.

Yet another reason could be that some proteins serve several functions, such as the efflux pump in pathogenic organisms which serve to pump toxic chemicals out from the inside of the cells to the external environment, but can also serve as a method to extricate antibiotics from the cell cytoplasm to the external environment [22, 23]. Thus, the Mar regulon may serve two functions, ie. extrication of bile and antibiotics.

### Genes associated with general metabolic mechanisms

The cold shock-like protein family, *cspE*, *cspC* and *cspB*, was significantly down-regulated in biofilm cells. Also, it was shown that the DNA protection and DNA binding genes, *STY4154* and *hupA*, respectively, were down-regulated. Many membrane transport proteins, such as *tatE*, were also down-regulated in the biofilm cells. These results showed that while the *S.* Typhi cells remained in the mature biofilm, the cells entered a state of stasis and reduce the energy requirements.

Ribosomal Modulation Factor (*rmf*) was the most down-regulated gene in the mature biofilm cells. Based on an article by Niven and El-Sharoud [24], *rmf* was used for stabilizing ribosomes during stress conditions; that is, it would be expected for *rmf* to be highly up-regulated during the early stage of biofilm formation. However, it can also be theorized that the biofilm cells collected in this study at 24 h post-incubation were already matured and have entered the stasis stage. Thus, to reduce or inhibit general protein translation in the cells, there was a need to down-regulate *rmf*. This hypothesis is supported by a paper published by Yamagishi et al. [25] that showed a reduction in *rmf* caused cells to lose their viability and ability to form ribosome dimers as well as when the cells transitioned from the growth to stationary state. It was also hypothesized in the paper that *rmf* is down-regulated under certain growth conditions.

This study found that ribosomal RNA (rRNA), such as multiple ribosomal subunit genes *rplD*, *rplW*, *rpsS* and *rplU*, were significantly up-regulated in the biofilm cells. This suggests that general protein translation continues even in the sessile biofilm tissue.

### Genes involved in biofilm regulation

*STY0893*, the *bssR* gene for biofilm regulation, was significantly down-regulated in the biofilm cells. According to the paper by Domka et al., [26], the *bssR* gene, also called *yliH* in *E. coli* cells, was up-regulated during biofilm formation. However, deletion of the *yliH* gene in *E. coli* caused the biofilm mass to increase by as much as 290-fold, but did not affect growth of the cells under normal conditions. However, this transcriptome study on *S.* Typhi biofilm cells showed that the *bssR* gene (*STY0893*) was significantly down-regulated as compared to the planktonic cells. BLAST analysis comparison between *E. coli* K-12 and *S.* Typhi CT18 showed that while the gene *bssR* is present in both species, it only has a 75% nucleotide similarity. Also since the base pair length is different between the two species; it can be assumed that the two proteins are different. The difference in results between this study (on *S*. Typhi) and the studies on *E. coli* [26] could be due to other parameters, such as different growth conditions, media and surfaces used for biofilm culture.

*YjfO* is a gene that encodes for biofilm stress and motility protein A. In a study done on *E. coli*, *yjfO* was found to be associated with cell survival by formation of biofilm in response to peroxide stress [27]. It was shown that *yjfO* was up-regulated in *E. coli* during the micro-colony formation and biofilm maturation processes. However, it was also shown to be unnecessary for planktonic cell growth or initial surface adhesion. Comparison of the results of the study by Weber et al. [27] with our data suggests that in *S.* Typhi there could possibly be a different function for the gene *yjfO*, as it was down-regulated in *S.* Typhi for biofilm formation. However, this can also be explained that since *yjfO* is responsible for micro-colony formation, it is also possible that the transcription rate of the gene in mature biofilm tissue was suppressed as there is no further need for the gene in the static environment of the biofilm tissue.

## Conclusion

This study provides an overview of the genes that are differentially expressed in *S.* Typhi when it transitioned from the planktonic to the mature biofilm phenotype. This transcriptome study identified 341 statistically significant (q < 0.05) genes associated with biofilm formation. KEGG pathway analysis showed that membrane genes associated with thickness and stickiness were up-regulated, presumably to allow the *S.* Typhi biofilm structure to stick to the surfaces and to each other to form mature biofilm. The results also showed that the mature biofilm cells entered into a sessile state accompanied by a down-regulation of genes involved in metabolic processes. However, the transcriptome of the mature biofilm state may not necessarily reflect the genetic changes needed to bring about the transition. For this, a transcriptome analysis should also be carried out on the intermediate (immature) biofilm cells in order to provide insights into the early stages of biofilm development. These data provided a basis for understanding the mechanisms of biofilm formation in *S*. Typhi and paved the way for discovery of novel genes or pathways associated with the development of the typhoid carrier state. These data may also be used to elucidate the effect of biofilm on the virulence and pathogenicity of *S*. Typhi and development of therapeutic agents designed to disrupt the process of biofilm formation.

## Additional files

Additional file 1: Table S1. Expression data for all genes found to be statistically significant. (DOCX 47 kb)

Additional file 2: Real time PCR (qPCR) was done on 8 selected genes, genes *tatE*, *STY1254*, *STY0893*, *rmf*, *lppA*, *opmC*, *yjfO*, and *lex*. The graphs in Figure S2 shows each gene and their real time PCR result based on relative fold change. Each gene’s relative fold change value reflects the log_2_-fold change result that was obtained using bioinformatics analysis. This validates the bioinformatics result as accurate. **Figure S2.** Relative fold-change in expression of the genes (A) *tatE*, (B) *STY1254*, (C) *STY0893*, (D) *RMF*, (E) *lppA*, (F) *opmC*, (G) *yjfO*, and (H) *lexA* of *S.* Typhi biofilm relative to planktonic cells. Statistics analysis (***P < 0.001, ****P < 0.0001, Student’s *t*-test, *n* = 3 independent experiments) using GraphPad Prism 6.0 software. Error bars represent SEM. **Table S2.1** Primer designed used for qPCR. (DOCX 70 kb)

## Abbreviations

NGS: Next generation sequencing
*Rmf*: Ribosomal modulation factor
rRNA: Ribosomal RNA
*S.* Typhi: *Salmonella enterica* subspecies *enterica* serovar Typhi
