# Supplementary material for: Transcriptomic study of Salmonella enterica subspecies enterica serovar Typhi biofilm
Source: BMC Genomics. 2017 Oct 31;18:836. doi: 10.1186/s12864-017-4212-6 (PMC5664820; doi:10.1186/s12864-017-4212-6)
Supplement: Supplementary file 1 — Expression data for all genes found to be statistically significant. (DOCX 47 kb) [file 12864_2017_4212_MOESM1_ESM.docx]

**Supplementary Data 1**

Supplementary Table S1: Expression data for all genes found to be statistically significant

| **No** | **Gene Name** | **Gene function** | **log_2_ fold change** | **p-value** | **q-value** |
| --- | --- | --- | --- | --- | --- |
| 1 | *STY1254* | hypothetical protein | 7.92174 | 5.00E-05 | 0.001837 |
| 2 | *STY1255* | hypothetical protein | 3.85549 | 4.50E-04 | 0.00998 |
| 3 | *yheA* | bacterioferritin-associated ferredoxin | 3.55635 | 2.00E-04 | 0.005683 |
| 4 | *rplD,rplW* | 50S ribosomal subunit protein L4, 50S ribosomal subunit protein L23 | 3.50218 | 7.00E-04 | 0.013329 |
| 5 | *STY3469* | hypothetical protein | 3.41257 | 5.00E-05 | 0.001837 |
| 6 | *priB* | 30s ribosomal subunit protein S18 | 3.25938 | 2.00E-04 | 0.005683 |
| 7 | *rpsS* | 50S ribosomal subunit protein L22 | 2.93416 | 5.00E-05 | 0.001837 |
| 8 | *STY4905* | hypothetical protein | 2.8976 | 5.00E-05 | 0.001837 |
| 9 | *rplU* | 50S ribosomal subunit protein L21 | 2.76352 | 5.00E-05 | 0.001837 |
| 10 | *rpmA* | 50S ribosomal subunit protein L27 | 2.74781 | 5.00E-05 | 0.001837 |
| 11 | *rpsJ* | 50S ribosomal subunit protein L3 | 2.65265 | 5.00E-05 | 0.001837 |
| 12 | *STY1229* | hypothetical protein | 2.60152 | 2.00E-04 | 0.005683 |
| 13 | *lexA* | LexA repressor | 2.58813 | 5.00E-05 | 0.001837 |
| 14 | *rplV* | 30S ribosomal subunit protein S3 | 2.53445 | 0.00015 | 0.004584 |
| 15 | *rpsI* | 30S ribosomal subunit protein S9 | 2.51349 | 5.00E-05 | 0.001837 |
| 16 | *rplR* | 30S ribosomal subunit protein S5 | 2.4115 | 5.00E-05 | 0.001837 |
| 17 | *rpsF* | 30s ribosomal protein S6 | 2.41089 | 5.00E-05 | 0.001837 |
| 18 | *yhdG* | tRNA-dihydrouridine synthase B | 2.38131 | 1.00E-04 | 0.003247 |
| 19 | *rplK* | 50S ribosomal subunit protein L11 | 2.35434 | 5.00E-05 | 0.001837 |
| 20 | *marA, marR* | multiple antibiotic resistance protein MarA, multiple antibiotic resistance protein MarR | 2.33256 | 1.05E-03 | 0.018538 |
| 21 | *pdhR* | pyruvate dehydrogenase complex repressor | 2.32856 | 5.00E-05 | 0.001837 |
| 22 | *rplM* | 50S ribosomal subunit protein L13 | 2.32687 | 1.35E-03 | 0.022217 |
| 23 | *STY4034* | putative IS1351 transposase (pseudogene) | 2.30975 | 0.00125 | 0.021145 |
| 24 | *ygbA* | hypothetical protein | 2.27833 | 5.00E-05 | 0.001837 |
| 25 | *yfhP* | DNA-binding transcriptional regulator IscR | 2.27551 | 0.00065 | 0.01271 |
| 26 | *rplA* | 50S ribosomal subunit protein L1 | 2.24644 | 0.00205 | 0.028899 |
| 27 | *hmpA* | flavohemoprotein | 2.19086 | 9.00E-04 | 0.016205 |
| 28 | *rpsN* | 30S ribosomal subunit protein S8 | 2.18809 | 5.00E-05 | 0.001837 |
| 29 | *rplB* | 30S ribosomal subunit protein S19 | 2.13394 | 2.00E-04 | 0.005683 |
| 30 | *yejG* | hypothetical protein | 2.06615 | 5.00E-05 | 0.001837 |
| 31 | *rplX* | 50S ribosomal subunit protein L5 | 1.98505 | 6.00E-04 | 0.012056 |
| 32 | *rplI* | 50s ribosomal subunit protein L9 | 1.9746 | 5.00E-05 | 0.001837 |
| 33 | *rplP,rpmC,rpsQ* | 50S ribosomal subunit protein L29, 30S ribosomal subunit protein S17, 50S ribosomal subunit protein L14 | 1.97287 | 0.00315 | 0.039918 |
| 34 | *rpmD* | 50S ribosomal subunit protein L15 | 1.95018 | 5.00E-05 | 0.001837 |
| 35 | *hslV* | heat shock protein | 1.91969 | 5.00E-05 | 0.001837 |
| 36 | *cvpA* | colicin V production protein | 1.91529 | 0.0014 | 0.022731 |
| 37 | *rplJ* | 50S ribosomal subunit protein L10 | 1.91146 | 5.00E-05 | 0.001837 |
| 38 | *rpsE* | 50S ribosomal subunit protein L30 | 1.9052 | 5.00E-05 | 0.001837 |
| 39 | *sefB* | fimbrial chaperone protein | 1.90357 | 0.0031 | 0.03956 |
| 40 | *STY3605* | hypothetical protein | 1.86259 | 5.00E-05 | 0.001837 |
| 41 | *fis* | Fis DNA-binding protein | 1.86108 | 5.00E-05 | 0.001837 |
| 42 | *rpsU* | 30S ribosomal subunit protein S21 | 1.82195 | 5.00E-05 | 0.001837 |
| 43 | *STY2355* | hypothetical protein | 1.81221 | 5.00E-05 | 0.001837 |
| 44 | *STY1389* | oxidoreductase | 1.79388 | 0.00365 | 0.044398 |
| 45 | *syd* | SecY interacting protein Syd | 1.75338 | 5.00E-05 | 0.001837 |
| 46 | *marB* | multiple antibiotic resistance protein MarB | 1.73274 | 5.00E-05 | 0.001837 |
| 47 | *STY3026,STY3027* | hypothetical protein, hypothetical protein | 1.71858 | 0.0018 | 0.027278 |
| 48 | *rpsG* | 30S ribosomal subunit protein S7 | 1.71652 | 0.00065 | 0.01271 |
| 49 | *STY4150,STY4151* | hypothetical protein, acetyltransferase | 1.71009 | 5.00E-04 | 0.010635 |
| 50 | *rplY* | 50s ribosomal protein L25 | 1.70593 | 5.00E-05 | 0.001837 |
| 51 | *rplN* | 50S ribosomal subunit protein L24 | 1.695 | 0.0016 | 0.024763 |
| 52 | *sprB* | AraC family transcriptional regulator | 1.68261 | 5.00E-05 | 0.001837 |
| 53 | *groES* | GroES protein | 1.67832 | 5.00E-05 | 0.001837 |
| 54 | *exbD* | biopolymer transport ExbD protein | 1.65929 | 0.00015 | 0.004584 |
| 55 | *sefR* | pseudogene | 1.65905 | 0.00015 | 0.004584 |
| 56 | *hslT* | heat shock protein B | 1.64875 | 0.00035 | 0.008375 |
| 57 | *apl* | phage regulatory protein | 1.64637 | 5.00E-05 | 0.001837 |
| 58 | *mgtC* | magnesium transport protein MgtC | 1.6427 | 5.00E-05 | 0.001837 |
| 59 | *sefA* | pseudogene | 1.63422 | 5.00E-05 | 0.001837 |
| 60 | *lipA* | lipoic acid synthetase | 1.63103 | 5.00E-05 | 0.001837 |
| 61 | *rplQ* | 50S ribosomal subunit protein L17 | 1.6138 | 1.00E-04 | 0.003247 |
| 62 | *STY3289* | hypothetical protein | 1.61019 | 0.00015 | 0.004584 |
| 63 | *STY3048* | hypothetical protein | 1.59606 | 5.00E-05 | 0.001837 |
| 64 | *exbB* | biopolymer transport ExbB protein | 1.58755 | 0.0017 | 0.025979 |
| 65 | *rpmG* | 50S ribosomal subunit protein L33 | 1.57956 | 9.00E-04 | 0.016205 |
| 66 | *STY2917* | transcriptional regulator | 1.57877 | 5.00E-05 | 0.001837 |
| 67 | *rplO* | 50S ribosomal subunit protein L15 | 1.56443 | 0.0012 | 0.02049 |
| 68 | *STY0014* | regulatory protein | 1.54511 | 5.00E-05 | 0.001837 |
| 69 | *tinR* | transcriptional regulator | 1.54328 | 5.00E-05 | 0.001837 |
| 70 | *rpmB* | 50S ribosomal subunit protein L28 | 1.51348 | 5.00E-05 | 0.001837 |
| 71 | *STY2918* | hypothetical protein | 1.50302 | 5.00E-05 | 0.001837 |
| 72 | *STY2676* | hypothetical protein | 1.49766 | 5.00E-05 | 0.001837 |
| 73 | *STY1514* | regulatory protein | 1.467 | 5.00E-05 | 0.001837 |
| 74 | *ssb* | single strand binding protein | 1.46499 | 5.00E-05 | 0.001837 |
| 75 | *STY0343* | pseudogene | 1.43219 | 0.0036 | 0.043937 |
| 76 | *STY3288* | hypothetical protein | 1.42763 | 5.00E-05 | 0.001837 |
| 77 | *ytfH* | transcriptional regulator | 1.42761 | 5.00E-05 | 0.001837 |
| 78 | *rbfA,truB* | ribosome-binding factor A, tRNA pseudouridine 55 synthase | 1.42428 | 0.00285 | 0.037152 |
| 79 | *trmD* | tRNA(guanine-N1)methyltransferase | 1.41603 | 0.00315 | 0.039918 |
| 80 | *yiiP* | transmembrane efflux protein | 1.41319 | 5.00E-04 | 0.010635 |
| 81 | *STY1391* | lipoprotein | 1.41087 | 5.00E-05 | 0.001837 |
| 82 | *gmk* | 5'guanylate kinase | 1.38379 | 0.00025 | 0.006495 |
| 83 | *rpsK* | 30S ribosomal subunit protein S11 | 1.37816 | 0.00055 | 0.011431 |
| 84 | *rpsM* | 30S ribosomal subunit protein S13 | 1.37448 | 0.00335 | 0.041726 |
| 85 | *STY3481* | hypothetical protein | 1.36263 | 5.00E-05 | 0.001837 |
| 86 | *STY4106* | lipoprotein | 1.36035 | 0.00195 | 0.028256 |
| 87 | *adk* | adenylate kinase | 1.35758 | 1.00E-04 | 0.003247 |
| 88 | *hscB* | chaperone protein HscB | 1.3541 | 5.00E-05 | 0.001837 |
| 89 | *STY3669* | hypothetical protein | 1.31702 | 0.0019 | 0.027977 |
| 90 | *sulA* | cell division inhibitor | 1.31245 | 0.00015 | 0.004584 |
| 91 | *STY2350* | hypothetical protein | 1.31008 | 7.00E-04 | 0.013329 |
| 92 | *STY0015* | hypothetical protein | 1.29943 | 5.00E-05 | 0.001837 |
| 93 | *STY4842,STY4843* | regulatory protein, GerE family regulatory protein | 1.29249 | 9.00E-04 | 0.016205 |
| 94 | *STY2359a* | pseudogene | 1.28997 | 0.00025 | 0.006495 |
| 95 | *yigF* | hypothetical protein | 1.28166 | 3.00E-04 | 0.007525 |
| 96 | *trxC* | thioredoxin 2 | 1.2792 | 0.00025 | 0.006495 |
| 97 | *STY4217* | pseudogene | 1.27443 | 0.0015 | 0.023515 |
| 98 | *hslR* | heat shock protein | 1.27079 | 0.00275 | 0.036238 |
| 99 | *nusG* | transcription antitermination protein | 1.26246 | 5.00E-05 | 0.001837 |
| 100 | *micF* | micF RNA post-transcriptionally regulates outer membrane protein F (OmpF) as a response to stess by binding and destabilising the ompF RNA message | 1.26223 | 0.0015 | 0.023515 |
| 101 | *rpmE* | 50S ribosomal protein L31 | 1.25663 | 0.00195 | 0.028256 |
| 102 | *STY4164* | LacI family transcriptional regulator | 1.2543 | 0.00025 | 0.006495 |
| 103 | *gpt* | xanthine-guanine phosphoribosyltransferase | 1.24343 | 0.00025 | 0.006495 |
| 104 | *STY0406* | DNA-binding transcriptional regulator | 1.23855 | 5.00E-05 | 0.001837 |
| 105 | *STY2872* | hypothetical protein | 1.23537 | 0.00045 | 0.00998 |
| 106 | *STY1102* | heat shock protein HspQ | 1.23269 | 0.0013 | 0.02159 |
| 107 | *ntpA* | DATP pyrophosphohydrolase | 1.2271 | 5.00E-04 | 0.010635 |
| 108 | *STY4535* | hypothetical protein | 1.22543 | 0.0015 | 0.023515 |
| 109 | *sodA* | manganese superoxide dismutase | 1.22229 | 5.00E-05 | 0.001837 |
| 110 | *yehE* | hypothetical protein | 1.21372 | 0.00045 | 0.00998 |
| 111 | *rpoE* | RNA polymerase sigma-E factor | 1.21317 | 0.0034 | 0.042061 |
| 112 | *STY4263* | hypothetical protein | 1.21296 | 5.00E-05 | 0.001837 |
| 113 | *rpsL* | 30S ribosomal subunit protein S12 | 1.1977 | 2.00E-04 | 0.005683 |
| 114 | *steA* | pseudogene | 1.19001 | 5.00E-05 | 0.001837 |
| 115 | *stgA* | fimbrial subunit | 1.18922 | 0.00035 | 0.008375 |
| 116 | *dnaJ* | DnaJ protein | 1.18263 | 0.00025 | 0.006495 |
| 117 | *smpA* | small protein A | 1.18061 | 0.00045 | 0.00998 |
| 118 | *rpoZ* | DNA-directed RNA polymerase subunit omega | 1.17833 | 7.00E-04 | 0.013329 |
| 119 | *STY0036* | LysR family transcriptional regulator | 1.17325 | 0.00255 | 0.034097 |
| 120 | *soxR* | redox-sensitivie transcriptional activator SoxR | 1.15534 | 1.00E-04 | 0.003247 |
| 121 | *tdk* | thymidine kinase | 1.15093 | 1.00E-04 | 0.003247 |
| 122 | *STY4003* | pseudogene | 1.14858 | 1.00E-04 | 0.003247 |
| 123 | *safA* | lipoprotein | 1.14429 | 0.00015 | 0.004584 |
| 124 | *STY3726a* | hypothetical protein | 1.1405 | 5.00E-04 | 0.010635 |
| 125 | *stpA* | DNA-binding protein StpA | 1.12944 | 0.00035 | 0.008375 |
| 126 | *stpA* | tyrosine phosphatase | 1.12944 | 0.00035 | 0.008375 |
| 127 | *STY0380* | outer membrane protein | 1.1213 | 1.00E-04 | 0.003247 |
| 128 | *creA* | hypothetical protein | 1.12064 | 1.00E-04 | 0.003247 |
| 129 | *rnhA* | ribonuclease H | 1.11132 | 0.00295 | 0.037779 |
| 130 | *STY3543* | hypothetical protein | 1.1061 | 6.00E-04 | 0.012056 |
| 131 | *invH* | cell adherance/invasion protein | 1.10579 | 6.00E-04 | 0.012056 |
| 132 | *STY1273* | hypothetical protein | 1.1047 | 0.0011 | 0.019142 |
| 133 | *STY3262* | hypothetical protein | 1.10395 | 0.00045 | 0.00998 |
| 134 | *STY1889* | hypothetical protein | 1.09673 | 0.00055 | 0.011431 |
| 135 | *STY3129* | N-acetylmuramoyl-L-alanine amidase | 1.0947 | 0.00025 | 0.006495 |
| 136 | *stgB* | fimbrial chaperone protein | 1.09408 | 0.00045 | 0.00998 |
| 137 | *STY1390* | transcriptional regulator | 1.08756 | 0.00025 | 0.006495 |
| 138 | *STY1513* | isomerase | 1.08646 | 0.00375 | 0.044864 |
| 139 | *csgB* | curlin monomer nucleation protein | 1.08508 | 0.0013 | 0.02159 |
| 140 | *rmbA* | hypothetical protein | 1.08251 | 7.00E-04 | 0.013329 |
| 141 | *yiiG* | lipoprotein | 1.08098 | 0.00055 | 0.011431 |
| 142 | *purF* | amidophosphoribosyltransferase | 1.07833 | 0.0039 | 0.046203 |
| 143 | *STY3618* | hypothetical protein | 1.0717 | 0.00045 | 0.00998 |
| 144 | *STY3880* | ATP/GTP-binding protein | 1.06989 | 3.00E-04 | 0.007525 |
| 145 | *STY3989* | carbohydrate kinase | 1.0672 | 1.00E-04 | 0.003247 |
| 146 | *STY1991* | acetyltransferase | 1.06543 | 3.00E-04 | 0.007525 |
| 147 | *ytfE* | iron-sulfur cluster repair di-iron protein | 1.06157 | 6.00E-04 | 0.012056 |
| 148 | *int* | bacteriophage integrase | 1.05547 | 0.00025 | 0.006495 |
| 149 | *int* | integrase | 1.05547 | 0.00025 | 0.006495 |
| 150 | *STY1439* | hypothetical protein | 1.05404 | 3.00E-04 | 0.007525 |
| 151 | *sipF* | acyl carrier protein | 1.05109 | 0.0011 | 0.019142 |
| 152 | *nemA* | N-ethylmaleimide reductase | 1.04588 | 0.00185 | 0.027463 |
| 153 | *STY1891* | pertussis-like toxin subunit | 1.04353 | 0.00125 | 0.021145 |
| 154 | *rplS* | 50S ribosomal subunit protein L19 | 1.04303 | 0.00275 | 0.036238 |
| 155 | *STY1359* | pseudogene | 1.04032 | 0.00025 | 0.006495 |
| 156 | *STY4461* | hypothetical protein | 1.03829 | 0.00115 | 0.019729 |
| 157 | *tnpA* | chitinase | 1.03513 | 0.00035 | 0.008375 |
| 158 | *tnpA* | insertion sequence element IS200 transposase | 1.03513 | 0.00035 | 0.008375 |
| 159 | *tnpA* | insertion sequence element IS200 transposase | 1.03513 | 0.00035 | 0.008375 |
| 160 | *tnpA* | lyase | 1.03513 | 0.00035 | 0.008375 |
| 161 | *tnpA* | hypothetical protein | 1.03513 | 0.00035 | 0.008375 |
| 162 | *tnpA* | hypothetical protein | 1.03513 | 0.00035 | 0.008375 |
| 163 | *tnpA* | pathogenicity island-encoded protein A | 1.03513 | 0.00035 | 0.008375 |
| 164 | *tnpA* | insertion sequence element IS200 transposase | 1.03513 | 0.00035 | 0.008375 |
| 165 | *tnpA* | insertion sequence element IS200 transposase | 1.03513 | 0.00035 | 0.008375 |
| 166 | *tnpA* | insertion sequence element IS200 transposase | 1.03513 | 0.00035 | 0.008375 |
| 167 | *tnpA* | pyruvate-flavodoxin oxidoreductase | 1.03513 | 0.00035 | 0.008375 |
| 168 | *tnpA* | insertion sequence element IS200 transposase | 1.03513 | 0.00035 | 0.008375 |
| 169 | *tnpA* | hypothetical protein | 1.03513 | 0.00035 | 0.008375 |
| 170 | *tnpA* | hypothetical protein | 1.03513 | 0.00035 | 0.008375 |
| 171 | *tnpA* | insertion sequence element IS200 transposase | 1.03513 | 0.00035 | 0.008375 |
| 172 | *tnpA* | insertion sequence element IS200 transposase | 1.03513 | 0.00035 | 0.008375 |
| 173 | *tnpA* | aminotransferase | 1.03513 | 0.00035 | 0.008375 |
| 174 | *tnpA* | NADP-dependent malate dehydrogenase | 1.03513 | 0.00035 | 0.008375 |
| 175 | *tnpA* | insertion sequence element IS200 transposase | 1.03513 | 0.00035 | 0.008375 |
| 176 | *tnpA* | insertion sequence element IS200 transposase | 1.03513 | 0.00035 | 0.008375 |
| 177 | *tnpA* | insertion sequence element IS200 transposase | 1.03513 | 0.00035 | 0.008375 |
| 178 | *tnpA* | insertion sequence element IS200 transposase | 1.03513 | 0.00035 | 0.008375 |
| 179 | *tnpA* | hypothetical protein | 1.03513 | 0.00035 | 0.008375 |
| 180 | *tnpA* | transposase | 1.03513 | 0.00035 | 0.008375 |
| 181 | *tnpA* | hypothetical protein | 1.03513 | 0.00035 | 0.008375 |
| 182 | *STY0381* | transcriptional regulator | 1.03212 | 0.00065 | 0.01271 |
| 183 | *yiaB* | hypothetical protein | 1.03042 | 6.00E-04 | 0.012056 |
| 184 | *atpH* | ATP synthase subunit delta | 1.02517 | 0.00065 | 0.01271 |
| 185 | *STY3029* | pseudogene | 1.02188 | 0.00075 | 0.014061 |
| 186 | *STY3169* | pseudogene | 1.00897 | 0.00035 | 0.008375 |
| 187 | *mutM* | formamidopyrimidine-DNA glycosylase | 1.00786 | 0.00035 | 0.008375 |
| 188 | *STY4903* | ferric iron reductase protein | 1.00778 | 5.00E-04 | 0.010635 |
| 189 | *dapA* | dihydrodipicolinate synthase | 0.999556 | 0.0014 | 0.022731 |
| 190 | *STY3538* | GntR family transcriptional regulator | 0.996384 | 2.00E-04 | 0.005683 |
| 191 | *stbA* | fimbrial protein | 0.995947 | 0.00225 | 0.030997 |
| 192 | *purH* | bifunctional phosphoribosylaminoimidazolecarboxamide formyltransferase/IMP cyclohydrolase; K00602 phosphoribosylaminoimidazolecarboxamide formyltransferase / IMP cyclohydrolase [EC:2.1.2.3 3.5.4.10] | 0.995165 | 0.0011 | 0.019142 |
| 193 | *STY4909* | dUMP phosphatase | 0.985829 | 0.00045 | 0.00998 |
| 194 | *yijD* | hypothetical protein | 0.984783 | 0.0025 | 0.033801 |
| 195 | *rpsP* | 30S ribosomal subunit protein S16 | 0.983957 | 0.002 | 0.028638 |
| 196 | *atpB* | ATP synthase subunit A | 0.982784 | 0.00025 | 0.006495 |
| 197 | *STY0756* | DNA recombinase | 0.976035 | 5.00E-04 | 0.010635 |
| 198 | *gidB* | glucose inhibited division protein | 0.975476 | 3.00E-04 | 0.007525 |
| 199 | *STY2366* | hypothetical protein | 0.97278 | 0.00235 | 0.032011 |
| 200 | *yafP* | acetyltransferase | 0.971473 | 7.00E-04 | 0.013329 |
| 201 | *coaD* | phosphopantetheine adenylyltransferase | 0.962028 | 0.00395 | 0.046492 |
| 202 | *STY4037* | pseudogene | 0.958791 | 5.00E-04 | 0.010635 |
| 203 | *glyQ* | glycine-tRNA synthetase subunit alpha | 0.957332 | 0.00135 | 0.022217 |
| 204 | *bcfA* | fimbrial subunit | 0.956545 | 0.00115 | 0.019729 |
| 205 | *STY4812* | acetyltransferase | 0.955472 | 0.0013 | 0.02159 |
| 206 | *fldB* | flavodoxin II | 0.953107 | 0.00105 | 0.018538 |
| 207 | *STY4610* | phage tail fiber protein | 0.950946 | 9.00E-04 | 0.016205 |
| 208 | *STY1867* | lipoprotein | 0.949275 | 0.00165 | 0.025321 |
| 209 | *STY3178* | hypothetical protein | 0.948244 | 0.00115 | 0.019729 |
| 210 | *STY2002* | hypothetical protein | 0.946715 | 9.00E-04 | 0.016205 |
| 211 | *yafB* | oxidoreductase | 0.934609 | 0.0015 | 0.023515 |
| 212 | *staA* | fimbrial protein | 0.930009 | 8.00E-04 | 0.014921 |
| 213 | *STY1950* | hypothetical protein | 0.929351 | 0.00255 | 0.034097 |
| 214 | *pipB* | secreted effector protein PipB | 0.920524 | 0.00255 | 0.034097 |
| 215 | *STY2364* | hypothetical protein | 0.917438 | 0.0022 | 0.03054 |
| 216 | *STY0843* | hypothetical protein | 0.914694 | 0.0016 | 0.024763 |
| 217 | *cigR* | pseudogene | 0.910417 | 0.00075 | 0.014061 |
| 218 | *STY4835* | pseudogene | 0.908981 | 0.0027 | 0.03597 |
| 219 | *sspB* | pathogenicity island 1 effector protein | 0.908033 | 0.00295 | 0.037779 |
| 220 | *sspB* | stringent starvation protein B | 0.908033 | 0.00295 | 0.037779 |
| 221 | *yrfE* | NUDIX hydrolase | 0.907985 | 0.0013 | 0.02159 |
| 222 | *STY0117* | hypothetical protein | 0.905455 | 0.00325 | 0.04076 |
| 223 | *tcfA* | fimbrial protein | 0.904386 | 0.00205 | 0.028899 |
| 224 | *asnC* | regulatory protein | 0.902997 | 0.00075 | 0.014061 |
| 225 | *STY4142* | lipoprotein | 0.902556 | 0.0028 | 0.036764 |
| 226 | *STY3821* | oligogalacturonate-specific porin protein | 0.89465 | 0.00185 | 0.027463 |
| 227 | *STY4526* | hypothetical protein | 0.894103 | 0.0014 | 0.022731 |
| 228 | *livJ* | pseudogene | 0.893961 | 0.00145 | 0.023335 |
| 229 | *ybaO* | transcriptional regulator | 0.893029 | 0.0016 | 0.024763 |
| 230 | *folA* | dihydrofolate reductase type I | 0.891433 | 0.00415 | 0.048222 |
| 231 | *ubiB* | flavin reductase | 0.888339 | 0.0037 | 0.044707 |
| 232 | *ubiC* | chorismate lyase | 0.887825 | 0.00165 | 0.025321 |
| 233 | *hemB* | delta-aminolevulinic acid dehydratase | 0.882724 | 0.00145 | 0.023335 |
| 234 | *dapB* | dihydrodipicolinate reductase | 0.881586 | 0.0021 | 0.029376 |
| 235 | *purT* | phosphoribosylglycinamide formyltransferase 2 | 0.862647 | 0.00355 | 0.043473 |
| 236 | *steD* | fimbrial subunit | 0.852885 | 0.0029 | 0.037669 |
| 237 | *STY3436* | carbohydrate kinase | 0.852159 | 0.00375 | 0.044864 |
| 238 | *STY3983* | pseudogene | 0.850769 | 0.00185 | 0.027463 |
| 239 | *STY0098* | pseudogene | 0.849002 | 0.00395 | 0.046492 |
| 240 | *yihV* | sugar kinase | 0.845247 | 0.00285 | 0.037152 |
| 241 | *STY0041* | hypothetical protein | 0.840736 | 0.00275 | 0.036238 |
| 242 | *marT* | pseudogene | 0.834822 | 0.0021 | 0.029376 |
| 243 | *yjfR* | L-ascorbate 6-phosphate lactonase | 0.833035 | 0.00205 | 0.028899 |
| 244 | *msrA* | peptide methionine sulfoxide reductase | 0.828487 | 0.00245 | 0.033249 |
| 245 | *STY3335* | AraC family transcriptional regulator | 0.82741 | 0.00235 | 0.032011 |
| 246 | *adiY* | AraC family transcriptional regulator | 0.815647 | 0.0034 | 0.042061 |
| 247 | *STY1388* | oxidoreductase | 0.797567 | 0.00375 | 0.044864 |
| 248 | *ydeW* | regulatory protein | 0.792668 | 0.00335 | 0.041726 |
| 249 | *atpE* | ATP synthase subunit C | 0.784174 | 0.0041 | 0.048102 |
| 250 | *STY4059* | hypothetical protein | 0.770436 | 0.00415 | 0.048222 |
| 251 | *agp* | glucose-1-phosphatase | -0.79275 | 0.00345 | 0.042391 |
| 252 | *STY2208* | DgsA anti-repressor MtfA | -0.86004 | 0.0039 | 0.046203 |
| 253 | *STY1628* | hypothetical protein | -0.86581 | 0.00295 | 0.037779 |
| 254 | *STY1416* | hypothetical protein | -0.86715 | 0.00215 | 0.02996 |
| 255 | *lrp* | leucine-responsive regulatory protein | -0.87274 | 0.002 | 0.028638 |
| 256 | *STY1252* | hypothetical protein | -0.88332 | 0.0037 | 0.044707 |
| 257 | *STY3504* | hypothetical protein | -0.89152 | 0.002 | 0.028638 |
| 258 | *yfeC* | hypothetical protein | -0.89343 | 0.0023 | 0.031566 |
| 259 | *yaeH* | hypothetical protein | -0.91959 | 0.00205 | 0.028899 |
| 260 | *yohC* | hypothetical protein | -0.92776 | 0.00195 | 0.028256 |
| 261 | *hupB* | DNA-binding protein HU-beta | -0.93753 | 0.0015 | 0.023515 |
| 262 | *STY1805* | hypothetical protein | -0.93983 | 0.00225 | 0.030997 |
| 263 | *caiF* | transcriptional activator CaiF | -0.95021 | 0.00045 | 0.00998 |
| 264 | *eutG,eutJ* | alchohol dehydrogenase, ethanolamine utilization protein EutJ | -0.9542 | 0.0032 | 0.040271 |
| 265 | *STY2722* | hypothetical protein | -0.95751 | 0.0032 | 0.040271 |
| 266 | *STY0929* | hypothetical protein | -0.96728 | 0.00045 | 0.00998 |
| 267 | *yehZ* | ABC transporter substrate-binding protein | -0.98175 | 0.00085 | 0.015693 |
| 268 | *eutE* | aldehyde dehydrogenase | -0.98344 | 0.00195 | 0.028256 |
| 269 | *slyA* | transcriptional regulator | -0.9941 | 4.00E-04 | 0.009509 |
| 270 | *slyA* | regulatory protein | -0.9941 | 4.00E-04 | 0.009509 |
| 271 | *STY0845* | pseudogene | -1.01377 | 0.00345 | 0.042391 |
| 272 | *yfeD* | hypothetical protein | -1.01751 | 0.0019 | 0.027977 |
| 273 | *yjfN* | hypothetical protein | -1.022 | 0.00295 | 0.037779 |
| 274 | *STY2213,STY2214* | hypothetical protein, hypothetical protein | -1.04297 | 0.00095 | 0.016937 |
| 275 | *STY3476* | RNA-binding protein YhbY | -1.04932 | 0.00415 | 0.048222 |
| 276 | *ybeJ* | ABC transporter substrate-binding protein | -1.0604 | 0.0038 | 0.045313 |
| 277 | *STY2542* | hypothetical protein | -1.08002 | 0.00095 | 0.016937 |
| 278 | *chaB* | cation transport regulator ChaB | -1.09019 | 0.00065 | 0.01271 |
| 279 | *ybdQ* | universal stress protein Usp | -1.11779 | 2.00E-04 | 0.005683 |
| 280 | *pspE* | phage shock protein E | -1.13601 | 6.00E-04 | 0.012056 |
| 281 | *STY3500* | sigma(54) modulation protein | -1.14679 | 2.00E-04 | 0.005683 |
| 282 | *STY3150* | lipoprotein | -1.16397 | 0.0015 | 0.023515 |
| 283 | *gyrI* | DNA gyrase inhibitory protein | -1.2162 | 1.00E-04 | 0.003247 |
| 284 | *STY1824* | methionine sulfoxide reductase B | -1.22257 | 5.00E-05 | 0.001837 |
| 285 | *STY0091* | lipoprotein | -1.22956 | 0.00055 | 0.011431 |
| 286 | *osmC* | osmotically inducible protein C | -1.27682 | 5.00E-05 | 0.001837 |
| 287 | *STY3365* | lipoprotein | -1.28008 | 5.00E-05 | 0.001837 |
| 288 | *psiF* | phosphate starvation-inducible protein PsiF | -1.29763 | 0.00035 | 0.008375 |
| 289 | *pduS* | ferredoxin | -1.34074 | 5.00E-05 | 0.001837 |
| 290 | *osmB* | osmotically inducible lipoprotein B | -1.34454 | 0.00185 | 0.027463 |
| 291 | *csrA* | carbon storage regulator | -1.3676 | 5.00E-05 | 0.001837 |
| 292 | *STY3123* | lipoprotein | -1.38337 | 5.00E-05 | 0.001837 |
| 293 | *ydhZ* | hypothetical protein | -1.3846 | 0.00175 | 0.026631 |
| 294 | *STY0509* | hypothetical protein | -1.42875 | 5.00E-05 | 0.001837 |
| 295 | *yceB* | hypothetical protein | -1.44838 | 0.00045 | 0.00998 |
| 296 | *bolA* | BolA protein | -1.46493 | 5.00E-05 | 0.001837 |
| 297 | *STY1154* | hypothetical protein | -1.48516 | 1.00E-04 | 0.003247 |
| 298 | *STY1846* | hypothetical protein | -1.51797 | 5.00E-05 | 0.001837 |
| 299 | *STY1031* | bacteriophage protein | -1.52387 | 5.00E-05 | 0.001837 |
| 300 | *ygaU* | pseudogene | -1.56911 | 5.00E-05 | 0.001837 |
| 301 | *sodB* | superoxide dismutase | -1.57203 | 5.00E-05 | 0.001837 |
| 302 | *STY3368a* | hypothetical protein | -1.57226 | 0.00015 | 0.004584 |
| 303 | *STY1823* | methionine sulfoxide reductase B | -1.61883 | 5.00E-05 | 0.001837 |
| 304 | *ssaH* | pathogenicity island protein | -1.66435 | 1.00E-04 | 0.003247 |
| 305 | *STY3408* | hypothetical protein | -1.68316 | 5.00E-05 | 0.001837 |
| 306 | *ybaJ* | Hha toxicity attenuator | -1.79603 | 0.00185 | 0.027463 |
| 307 | *yaiB* | anti-RssB factor | -1.80423 | 5.00E-05 | 0.001837 |
| 308 | *STY1875* | lysozyme inhibitor | -1.82335 | 5.00E-05 | 0.001837 |
| 309 | *ytfK* | hypothetical protein | -1.83414 | 5.00E-05 | 0.001837 |
| 310 | *yodD* | hypothetical protein | -1.87839 | 1.00E-04 | 0.003247 |
| 311 | *manX* | PTS system mannose-specific transporter subunit IIAB | -1.88781 | 5.00E-05 | 0.001837 |
| 312 | *tdcA* | TDC operon transcriptional activator | -1.88951 | 0.00025 | 0.006495 |
| 313 | *STY1124* | hypothetical protein | -1.93662 | 5.00E-05 | 0.001837 |
| 314 | *STY1856* | regulatory protein | -2.07402 | 5.00E-05 | 0.001837 |
| 315 | *yiiU* | FtsZ stabilizer | -2.10309 | 5.00E-05 | 0.001837 |
| 316 | *cspB* | cold shock protein | -2.18312 | 5.00E-05 | 0.001837 |
| 317 | *STY0398* | propionate catabolism operon regulatory protein | -2.29804 | 5.00E-05 | 0.001837 |
| 318 | *STY0788* | hypothetical protein | -2.35255 | 5.00E-05 | 0.001837 |
| 319 | *osmE* | osmotically inducible lipoprotein E | -2.36034 | 5.00E-05 | 0.001837 |
| 320 | *tatE* | sec-independent protein translocase protein TatE | -2.41698 | 5.00E-05 | 0.001837 |
| 321 | *ompX* | outer membrane protein X | -2.45886 | 2.00E-04 | 0.005683 |
| 322 | *yaiA* | hypothetical protein | -2.48198 | 5.00E-05 | 0.001837 |
| 323 | *hupA* | histone like DNA-binding protein HU-alpha | -2.59176 | 5.00E-05 | 0.001837 |
| 324 | *STY1982* | hypothetical protein | -2.61628 | 5.00E-05 | 0.001837 |
| 325 | *STY1323* | hypothetical protein | -2.67081 | 5.00E-05 | 0.001837 |
| 326 | *ompC* | outer membrane protein C | -2.75117 | 5.00E-05 | 0.001837 |
| 327 | *lppA* | major outer membrane lipoprotein | -2.93078 | 5.00E-05 | 0.001837 |
| 328 | *STY1938* | hypothetical protein | -3.30784 | 5.00E-05 | 0.001837 |
| 329 | *rpsV* | 30S ribosomal protein S22 | -3.30884 | 5.00E-05 | 0.001837 |
| 330 | *yjfO* | biofilm stress and motility protein A | -3.3313 | 5.00E-05 | 0.001837 |
| 331 | *cspC* | cold shock-like protein CspC | -3.34324 | 5.00E-05 | 0.001837 |
| 332 | *STY2264* | hypothetical protein | -3.38655 | 5.00E-05 | 0.001837 |
| 333 | *dps* | DNA protection during starvation protein | -3.42148 | 5.00E-05 | 0.001837 |
| 334 | *STY4154* | DNA-binding protein | -3.45538 | 0.00085 | 0.015693 |
| 335 | *ecnB* | entericidin B | -3.5082 | 5.00E-05 | 0.001837 |
| 336 | *STY0800* | hypothetical protein | -3.80587 | 5.00E-05 | 0.001837 |
| 337 | *STY1854* | hypothetical protein | -4.01921 | 5.00E-05 | 0.001837 |
| 338 | *cspE* | cold shock-like protein cspE | -4.36039 | 5.00E-05 | 0.001837 |
| 339 | *STY4436* | hypothetical protein | -4.50574 | 5.00E-05 | 0.001837 |
| 340 | *STY0893* | biofilm formation regulatory protein BssR | -5.12497 | 5.00E-05 | 0.001837 |
| 341 | *rmf* | ribosome modulation factor | -6.30581 | 5.00E-05 | 0.001837 |
