# Supplementary material for: Transcriptomic study of Salmonella enterica subspecies enterica serovar Typhi biofilm
Source: BMC Genomics. 2017 Oct 31;18:836. doi: 10.1186/s12864-017-4212-6 (PMC5664820; doi:10.1186/s12864-017-4212-6)
Supplement: Supplementary file 2 — Real time PCR (qPCR) was done on 8 selected genes, genes tatE, STY1254, STY0893, rmf, lppA, opmC, yjfO, and lex. The graphs in Figure S2 shows each gene and their real time PCR result based on relative fold change. Each gene’s relative fold change value reflects the log2-fold change result that was obtained using bioinformatics analysis. This validates the bioinformatics result as accurate. Figure S2. Relative fold-change in expression of the genes (A) tatE, (B) STY1254, (C) STY0893, (D) RMF, (E) lppA, (F) opmC, (G) yjfO, and (H) lexA of S. Typhi biofilm relative to planktonic cells. Statistics analysis (***P < 0.001, ****P < 0.0001, Student’s t-test, n = 3 independent experiments) using GraphPad Prism 6.0 software. Error bars represent SEM. Table S2.1 Primer designed used for qPCR. (DOCX 70 kb) [file 12864_2017_4212_MOESM2_ESM.docx]

**Supplementary data 2**

Real time PCR (qPCR) was done on 8 selected genes, genes *tatE*, *STY1254*, *STY0893*, *rmf*, *lppA*, *opmC*, *yjfO*, and *lex*. The graphs in figure S2 shows each gene and their real time PCR result based on relative fold change. Each gene’s relative fold change value reflects the log_2_-fold change result that was obtained using bioinformatics analysis. This validates the bioinformatics result as accurate.

**B**

**A**

**D**

**C**

**F**

**E**

**H**

**G**

Figure S2: Relative fold-change in expression of the genes (A) *tatE*, (B) *STY1254*, (C) *STY0893*, (D) *RMF*, (E) *lppA*, (F) *opmC*, (G) *yjfO*, and (H) *lexA* of *S.* Typhi biofilm relative to planktonic cells. Statistics analysis (***P<0.001, ****P<0.0001, Student’s t-test, n=3 independent experiments) using GraphPad Prism 6.0 software. Error bars represent SEM.

Table 2.1: Primer designed used for qPCR.

| **No.** | **Gene** | **Forward primer** | **Reverse primer** |
| --- | --- | --- | --- |
| 1 | *invA* | GTGAAATTATCGCCACGTTCGGGCAA | TCATCGCACCGTCAAAGGAACC |
| 2 | *STY1254* | GCCAGAAGGTCAACAGAAGT (Sense) | CGGTAACAGAGGTAATACGGAAAG (AntiSense) |
| 3 | *rmf* | CTGGAACGGGCACATCAA (Sense) | CCAACCTCCCAACCAGTATG (AntiSense) |
| 4 | *yjfO* | CGTCGGTTTGCTCCATTGATA (Sense) | GCTGTAGTCCTTGCGTTTGA (AntiSense) |
| 5 | *tatE* | TTGCCGCACTGGTTGTT (Sense) | CCGCGTCTTCATCGTTCAT (AntiSense) |
| 6 | *STY0893* | GACAGACTGAGAACCGATCTTC (Sense) | TTTCGCTGACTGACATATACCC (AntiSense) |
| 7 | *lexA* | CATTACCAGGTCGATCCTTCAC (Sense) | TATGTACCGCCAGCAAATCC (AntiSense) |
| 8 | *ompC* | GCGCTATCACCACGTCTAAA (Sense) | AATACTGCGCTGCCAGATAG (AntiSense) |
| 9 | *lppA* | TGCTCCAGCAACGCTAAA (Sense) | TCGTCTTTAGCAGCCTGAAC (AntiSense) |
